# Explaining gender inequalities in overweight people: a Blinder-Oaxaca decomposition analysis in northern Sweden

**DOI:** 10.1186/s12939-023-01973-9

**Published:** 2023-08-22

**Authors:** Fethi Mohammed Yusuf, Miguel San Sebastián, Masoud Vaezghasemi

**Affiliations:** Department of Epidemiology and Global Health, Umeå University, Umeå, SE -901 87 Sweden

**Keywords:** Sweden, Overweight, Obesity, Gender, Inequalities, Oaxaca decomposition

## Abstract

**Background:**

Being overweight and obesity are considered serious public health concerns worldwide. At the population level, factors contributing to overweight as well as the differences in overweight between men and women in terms of prevalence or associated factors are relatively well-known. What is less known is what explains the inequalities in overweight between men and women. In this study, we examined the contribution of material, behavioural, and psychosocial factors in explaining the gender differences in overweight among adults in northern Sweden.

**Methods:**

This study was based on the 2018 Swedish Health on Equal Terms survey, which was carried out in Sweden’s four northernmost regions. The analytical sample consisted of 20,855 participants (47% men) aged 20–84 years. Overweight (including obesity) was the outcome, and the selected explanatory variables were grouped according to three theoretical perspectives: material, behavioural and psychosocial. Descriptive statistics and Blinder-Oaxaca decomposition were applied for analysing the data.

**Results:**

Our study showed that the prevalence of overweight was 64% and 52% among men and women, respectively. It, therefore, revealed a gender gap in overweight people of 11.7% points with explanatory factors accounting for 39% of that gap. This gender gap in overweight people was mostly explained by behavioural variables (19.3%), followed by the materialistic variables and age accounting for 16.2% and 3.1%, respectively. Specifically, having low education, being in the lowest income quintile, alcohol drinking and snus usage contributed to explain 8.4%, 8.9%, 2.8% and 6.3% of the gender difference, respectively.

**Conclusions:**

We found a considerable gender inequality in overweight between men and women. The findings highlight that future overweight prevention initiatives would benefit from targeting the uncovered contributing factors to reduce gender inequalities in overweight people.

## Background

Obesity is one of the most serious public health challenges of the 21st century, placing adults at increased risk of morbidity and mortality. In 2015, high body mass index (BMI) caused an estimated 4 million deaths and 40 million disability-adjusted life years (DALY) among adults worldwide [1]. Excess weight lowers the quality of life, increases medical costs, strains the healthcare system and results in productivity losses [2]. In addition, high BMI is recognised as the main risk factor for several chronic conditions such as type 2 diabetes, cardiovascular diseases, osteoarthritis, hepatitis and some cancers [3, 4].

In the last three decades, overweight population has grown dramatically [5]. According to a World Health Organization report, there were more than 1.9 billion overweight adults in 2016 globally [5]. In the case of the European region, many countries have an overweight prevalence of around 50% [6, 7]. For instance, in Sweden, despite increasing calls for action, there has also been a rising trend in the prevalence of overweight and obese people. Between 1995 and 2017, the prevalence of obesity and overweight increased by 86% and 23%, respectively, while severe obesity increased by 153% [8]. The rise in overweight and obesity rates is increasingly considered to be the result of growing social inequalities and changes in lifestyle behaviour such as dietary habits, smoking and alcohol consumption [9].

The prevalence of overweight and obesity appears to differ by gender, as significant sex differences in overweight and obesity across countries are consistently shown in the literature [1, 10]. According to a review of 68 countries by Wells et al., there were 3 obese women for every 2 obese men [11]. Other studies also reinforce the finding that women frequently show a higher rate of obesity [12–14]. Gender differences in diet, physical exercise and lifestyle factors are potentially generating these gender inequalities in overweight/obesity risk [15–18]. These gender variations may be due to differences in socioeconomic indicators like income and education between men and women [19].

In Sweden, the prevalence of overweight and obesity is higher among men compared to women [8]. Hence, it is worth investigating why these differences in overweight between men and women exist in order to (i) provide direction for research and preventive policy and (ii) to reduce inequalities in excess weight between men and women.

Previous research in the field has mainly focused on identification of factors associated with overweight and obesity among men and women separately [20, 21]. To our knowledge, very little is known about what might explain the gender gap. This study, therefore, sought (i) to estimate the gender gap of overweight people as well as (ii) to explain inequalities in overweight between men and women in northern Sweden.

## Methods

### Study design and data collection

This study used the most recent secondary data from the 2018 Health on Equal Terms (HET) cross-sectional survey carried out in the four northernmost regions (Västernorrland, Jämtland, Västerbotten and Norrbotten) of northern Sweden. The survey represents the regionally expanded sample of the national HET survey, which is implemented as a collaboration between the Swedish National Public Health Agency and the individual regions, with the purpose of monitoring the health and living conditions of the population. All residents aged 20–84 years in the aforementioned four regions were identified as the target population, and a random sample stratified by age, gender, region and municipality was selected. The survey gathered information from 23,487 respondents who answered either the postal or web questionnaire representing a 58.6% response rate. The survey data was linked to individual-level register data on, for example, income and education, through the Swedish Personal Identity Number.

### Measures

The outcome variable in this study was whether an individual was or was not overweight (including obese). BMI was computed from self-reported weight and height data and calculated as weight (kg) divided by height squared (m^2^). BMI categories were calculated in accordance with World Health Organization guidelines [22]. Overweight was defined as having a BMI ≥ 25 kg/m^2^. In our analysis, we considered both overweight and obese categories within the same group. Gender was used as the exposure variable, and participants were grouped into men and women based on the registered data. Explanatory variables with a plausible link to overweight were broadly categorised into three theoretical perspectives: materialistic, behavioural and psychosocial (adapted from the conceptual framework on social determinants of health) [23].

The age of the participants was categorised into 20–60 years (reference) and 61–84 years. The material variables included education and income. Education was categorised into high (three or more years of tertiary education, reference), medium (up to two years of tertiary education) and low (less than three years of secondary education). Income was based on the individual disposable income and coded into five quintiles, with the first quintile (reference) being the highest one. Disposable income is defined here as the amount of money (Swedish Krona) available to be spent or saved at discretion, after deducting taxes and social security charges.

Behavioural variables included smoking, use of snus (the Swedish moist tobacco product), risky alcohol drinking, vegetable and fruit intake and physical inactivity. Smoking was coded as current smoker or not while snus usage was coded as yes/no. Risky alcohol drinking was captured by Audit-C [24], which comprises the following three questions : ‘How often did you drink alcohol?’ with response options ranging from 0 (never) to 4 (four times a week or more often), ‘How many ‘glasses’ did you drink on a typical day when you drank alcohol?’ with response options ranging from 0 (1–2) to 4 (10 or more), and ‘How often did you drink six ‘glasses’ or more at a time in the past 12 months?’ with response options ranging from 0 (never) to 4 (daily or almost daily). The maximum total score for Audit-C is 12 and is calculated by summing up the scores for all items. The cut-off point for risky drinking was determined as > 5 for men, and > 4 for women [24]. Vegetable and fruit consumption was operationalised using the questions: ‘How often do you eat vegetables and root vegetables?’ and ‘How often do you eat fruits and berries?’ The two variables were added and dichotomized into daily (if both vegetables and fruits were consumed each day) and not daily. Physical inactivity was captured by the following question ‘How much do you sit during a normal day, not counting sleep?’ and was coded as ≤ 6hr/day and > 6hr/day. Finally, stress was added as part of the psychosocial model and measured using the following question ‘Do you feel stressed at present?’ with response options as not at all, to some extent, quite a lot, and very much. These answers were further dichotomised into No for ‘not at all’ with all other answers deemed as Yes.

### Data analysis

Descriptive statistical analysis was done to summarise the proportion of overweight people in total and by sex across the explanatory variables. Then, the Blinder-Oaxaca decomposition analysis was conducted to estimate and decompose the disparity in overweight between men and women. The Blinder-Oaxaca decomposition approach allows for a comparison between two groups (men and women) of variables [25, 26], producing an explained component that corresponds to the contribution to the inequality by the differences in the included variables and a residual component, which corresponds to what cannot be explained. The significance level was set at a p-value of less than 0.05. The variance inflation factor (VIF) was used to check for multicollinearity (mean VIF value was 1.1). A complete case analysis was applied to handle missing data which accounted for 7% of the collected data. Therefore, 20,855 (93%) of the observations (9,721 men and 11,134 women) were included in the analysis.

### Ethics consideration

All participants provided informed consent for the use of the data for research purposes. The study was approved by the Swedish Ethical Review Authority (reference number 2020–00204 and 2015/134–31).

## Results

### Descriptive statistics

Table 1 summarises the study sample’s characteristics by gender. Most participants were in the age group below 60 years and had attained less than 3 years of secondary education. Men were more likely than women to report using alcohol and snus. Additionally, most men did not regularly eat fruits and vegetables and were physically sedentary for more than six hours every day. In contrast, women reported smoking and stress at higher levels than men.

The prevalence of overweight was 64% and 52% among men and women, respectively. As education level increased, the prevalence of overweight declined. Overweight among men steadily decreased along income quintiles, but a similar trend was not seen among women. More than half of both men and women who did not regularly consume vegetable and fruit and were physically inactive for > 6 h/day were overweight (Table 1).

**Table 1:** Distribution of background characteristics of participants and prevalence of overweight by risk factors

Characteristic	Men (N %)	Women N (%)	Overweight men N (%)	Overweight women N (%)
Age group				
≤ 60 years	4812 (49)	6130 (55)	2987 (62)	2957 (48)
> 60 years	4909 (51)	5004 (45)	3294 (67)	2925 (58)
**Material**				
Education				
High	1646 (17)	2951 (26)	893 (54)	1282 (43)
Medium	3656 (38)	3857 (35)	2249 (62)	1975 (51)
Low	4419 (45)	4326 (39)	3139 (71)	2625 (61)
Income				
Highest	1944 (20)	2227 (20)	872 (58)	1416 (53)
Quintile 2	1944 (20)	2227 (20)	1170 (63)	1285 (56)
Quintile 3	1944 (20)	2227 (20)	1120 (67)	1346 (54)
Quintile 4	1944 (20)	2227 (20)	1340 (67)	1108 (51)
Lowest	1945 (20)	2227 (20)	1779 (66)	727 (49)
**Behavioural**				
Current Smoking				
No	9212 (95)	10,364 (93)	5973 (65)	5463 (53)
Yes	509 (5)	770 (7)	308 (61)	419 (54)
Snus				
No	6474 (67)	9934 (89)	4011 (62)	5291 (53)
Yes	3247 (33)	1200 (11)	2270 (70)	591 (49)
Risky alcohol drink				
No	8147 (84)	10,048 (90)	5148 63)	5285 (53)
Yes	1574 (16)	1086 (10)	1133 (72)	597 (55)
Vegetable and fruit consumption				
Daily	4798 (49)	7523 (68)	2982 (62)	3853 (51)
Not daily	4923 (51)	3611 (32)	3299 (67)	2029 (56)
Physical inactivity				
≤ 6 h/day	5588 (57)	7123 (64)	3540 (63)	3649 (51)
> 6 h/day	4133 (43)	4011 (36)	2741 (66)	2233 (56)
**Psychosocial**				
Stress				
No	5519 (57)	5265 (47)	3616 (65)	2840 (54)
Yes	4202 (43)	5869 (53)	2665 (63)	3042 (52)

### Decomposition analysis

**Fig. 1:**
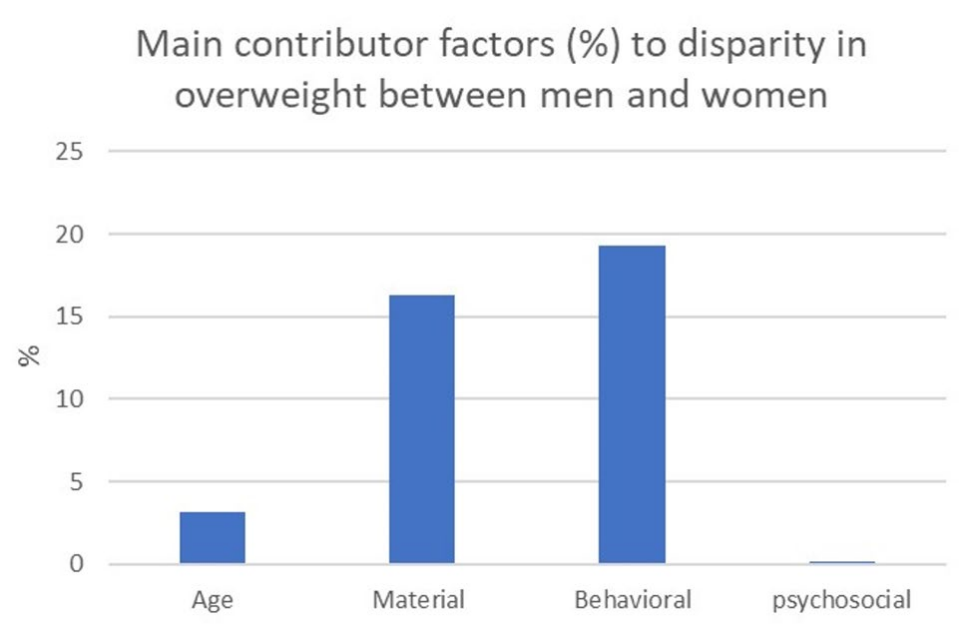
Relative (%) contributions of the groups of variables used in the decomposition analysis

Overall, the men-women inequality in overweight people was estimated to be 11.7% points (Table 2). Of this difference, 39% was explained by the selected variables while the rest remained unexplained. Regarding age, to be in the 61–84 years group explained 3.1% of the gender gap in overweight people. Among the materialistic factors, low education and being in the 5th quintile explained 8.4% and 8.9% of the inequality in overweight people, respectively. All the included behavioral factors contributed to explaining the gap in overweight people. Snus and vegetable and fruit intake contributed to the inequality by 6.3% and 6.7%, respectively. However, stress did not play any role in explaining the inequality (Table 2). Figure 1 depicts the material, behavioural and psychosocial contributions to explaining the gender inequality in overweight people.

**Table 2:** Decomposition of the difference in overweight between men and women in northern Sweden

Prevalence of overweight among men			64%	
Prevalence of overweight among Women			52%	
Men- Women difference			11.7% points	
Total explained Difference			4.6% points	
Total unexplained Difference			7.1% points	
**Variables**	**coefficient**		**Contribution (%)**	**p-value**
Age group (ref: ≤60 years)				
> 60 years		0.0037	3.1%	< 0.001
**Material**				
Education level (ref: High)				
Medium	0.0021		1.7%	< 0.001
Low	0.010		8.4%	< 0.001
Income (ref: Highest)				
Quintile 2	-0.0004		-0.3%	0.95
Quintile 3	-0.0034		-2.8%	< 0.05
Quintile 4	0.0005		0.4%	< 0.001
Lowest	0.010		8.9%	< 0.001
**Behavioural**				
Current smoking (ref: No)				
Yes	0.0007		0.5%	< 0.05
Snus (ref: No)				
Yes	0.0075		6.3%	< 0.001
Risky alcohol drink (ref: No)				
Yes	0.0033		2.8%	< 0.001
Consumption of vegetable/fruit (ref: Daily)				
Not daily	0.008		6.7%	< 0.001
Physical inactivity (ref: ≤ 6 h/day)				
> 6 h/day	0.0035		2.9%	< 0.001
**Psychosocial**				
Stress (ref: No)				
Yes	0.0002		0.1%	0.73

## Discussion

This study estimated the gender inequality of overweight people in northern Sweden, using data from the most recent HET survey. It revealed a gap of 11.7% points with material, behavioural and psychosocial factors accounting for 39% of the gap. Education, income, snus use and diet accounted for most of the explained inequality (Table 2).

Our study showed that age was an important contributor to explaining the gender gap in overweight people, but the degree of inequality varied across both age groups. Men over the age of 60 exhibited a higher prevalence of overweight in comparison to women. The observed gap could be explained by a decreased physical activity and poorer diet among older men, maybe due to poor cooking skills and low motivation to change eating habits [27]. Similar results have been found in other studies that linked age to being overweight [7, 10, 28].

Correspondingly, education had a relevant role in explaining the gender inequality in overweight people. In this study, men with low and medium education were more likely than women to be overweight. Individual’s education attainment generally ensures acquisition of health promoting behaviours. Hence, highly educated women might be better aware of the consequences of obesity and more likely to engage in preventive health behaviours like regular exercise than men [29].

Furthermore, our study showed that overweight affects men and women with different income in a distinct degree. Generally, men were more likely to be overweight than women across income quintiles with the largest gender inequality in overweight people found within the lowest quintile. Socioeconomic differences in diet/nutrition and leisure-time physical activity might explain the above observed gender gap. Women might be more likely than men to spend their income on healthier diets and weight reduction activities over obesogenic foods and drinks such as alcohol. Prior studies also reinforce the aforementioned finding [27, 30]. Overweight and obese people, on the other hand, frequently experience less favourable material circumstances, including lower incomes and less opportunities for employment due to discrimination [31]. Thus, their ability to buy healthy food could be greatly impacted by their lower income, which ultimately leads to weight gain [32].

Similarly, our study showed that men snus users were more likely than women to be overweight. Use of snus might be a marker of other unhealthy behaviours, such as alcohol and food habits. Though several studies have explored the association between snus use and weight, but the findings are not conclusive. Some reported results similar to ours [33, 34], while other studies found no differences in mean BMI [35, 36]. Since little is known about the metabolic effects of snus use, more research is needed to explore the possible role of snus in overweight.

We also found certain gender differences in alcohol use that potentially generated gender inequalities in overweight people. Alcohol provides calories in addition to those obtained from other foods, which can lead to a positive energy balance and weight gain. The findings were consistent with prior studies, where heavy drinking was associated with a higher BMI [33, 37–39].

Moreover, our study showed that particularly diet and less physical activity contributed to the gender gap in overweight people. The consumption of a healthy diet, like vegetables and fruits, reduces weight gain, which was observed primarily among women. It is also possible that women who eat healthier foods are more likely to engage in health promoting behaviours like exercise and avoiding alcoholic beverages [37]. On the other hand, decreased energy expenditure from physical inactivity results in weight gain, which was seen mainly among men in our study. This can be supported by several studies that have shown dietary behaviour and exercise to be significantly associated with BMI [30, 34, 40, 41].

### Methodological considerations

To our knowledge, very little is known about the factors explaining the gender inequalities in overweight and obesity globally, as the majority of previous studies conducted separate analyses for men and women. The large population-based sample, which was linked with registered data on gender, age, education and income, can be considered strengths of this study. Hence, reported bias on such key register-based variables is expected to be reduced.

This study also has some limitations that need to be taken into account when interpreting the results. The study used a cross-sectional design, which implies that the study identified factors that contribute to inequality in overweight people but cannot provide causal inference. Second, weight and height were self-reported which could possibly result in biased estimates of the prevalence of overweight in the general population because of potential issues of social desirability. However, our rates of overweight were comparable to those found in another study conducted in Sweden, using objective measurements of body weight and height [8]. Likewise, the data on tobacco smoking, snus use, and alcohol habits were self-reported, which may have led to underreporting, the extent of this bias is however impossible to estimate. Another drawback of the study is that it only explained 39% of the disparity in overweight, leaving many other potential explanatory factors unknown. Future studies should be specifically designed to disentangle the gender inequality in terms of being overweight.

## Conclusions

We found a high prevalence of overweight in both sexes and a considerable inequality in overweight between men and women in northern Sweden. Our study also revealed that low education and income, as well as poor lifestyle factors, were the most important factors explaining the gender differences in overweight prevalence. The findings indicate that future overweight prevention initiatives would benefit from targeting the uncovered contributing factors to reduce the gender inequalities in overweight people.

## Data Availability

The data that support the findings of this study are available from Norrbotten, Västerbotten, Västernorrland and Jämtland/Härjedalen regions, but these data are not publicly available. However, data are available from the corresponding author upon reasonable request and with permission of the four regions.

## Abbreviations

BMI: Body mass index
DALY: Disability adjusted life year
HET: Health on Equal Terms
VIF: Variance inflation factor
